# Paget-Schroetter Syndrome With Focal Subclavian Arterial Stenosis Following Repetitive Overhead Activity: A Case Report

**DOI:** 10.7759/cureus.117355

**Published:** 2026-10-02

**Authors:** Badria Tebaig, Aiman Balouch, Mahboob Ur Rahman, Muhammad Asad Raza

**Affiliations:** 1 Acute Medicine, NHS Humber Health Partnership, Northern Lincolnshire, GBR; 2 Acute Medicine, Northern Lincolnshire and Goole NHS Foundation Trust, Scunthorpe, GBR; 3 Acute Medicine, Hull Royal Infirmary, Scunthorpe, GBR

**Keywords:** effort thrombosis, first rib resection, paget-schroetter syndrome, subclavian vein thrombosis, thoracic outlet syndrome, venous thrombosis

## Abstract

Paget-Schroetter syndrome (PSS), also known as effort thrombosis, is a rare form of upper extremity deep vein thrombosis resulting from mechanical compression of the subclavian vein at the thoracic outlet. It typically occurs in young, active individuals performing strenuous upper limb exercises and presents with pain, swelling, and discoloration of the affected limb. Early diagnosis and specialist management are important for optimizing outcomes. In selected patients, management may include a combined endovascular and surgical approach to restore venous patency and reduce the risk of recurrence and post-thrombotic complications.

A 46-year-old woman who was neither an athlete nor a manual laborer presented with a four-day history of right upper-limb pain and swelling following repetitive overhead activity. She reported progressive swelling, positional bluish discoloration on arm elevation, and episodic pallor of the thumb and index finger. No secondary provoking factor for venous thromboembolism was identified. Physical examination revealed a swollen right arm with prominent superficial veins and bluish discoloration provoked within 10-15 seconds of arm elevation. Initial duplex ultrasonography identified right subclavian vein thrombosis, with the axillary and brachial veins reported as patent. However, computed tomography (CT) venography performed the following day demonstrated thrombosis involving both the right axillary and subclavian veins. CT angiography additionally demonstrated focal stenosis of the right subclavian artery at the thoracic outlet. The patient underwent catheter-directed thrombolysis followed by first rib resection for thoracic outlet decompression.

This case documents PSS outside its typical demographic in a patient presenting with upper limb swelling and discoloration following repetitive overhead activity. The discordant axillary vein findings emphasize the importance of interpreting sequential vascular imaging studies in the context of their timing and modality. The accompanying episodic digital pallor and CT finding of focal subclavian arterial stenosis highlight the importance of considering possible arterial involvement in a predominantly venous presentation. Following treatment, the symptoms resolved, and venous patency was maintained at four-week follow-up.

## Introduction

Thoracic outlet syndrome encompasses neurogenic, venous and arterial disorders caused by compression of the brachial plexus, subclavian vein or subclavian artery, respectively, as these structures traverse the thoracic outlet [1,2]. Paget-Schroetter syndrome (PSS) is a form of primary axillo-subclavian deep vein thrombosis (DVT) associated with repetitive mechanical compression of the subclavian vein within the costoclavicular space, where the vein passes over the first rib, beneath the clavicle and anterior to the anterior scalene muscle. Repetitive or strenuous upper-limb activity may contribute to endothelial injury, venous stasis and thrombus formation [1-3].

PSS is rare, with a reported annual incidence of approximately 1-2 cases per 100,000 population, and most commonly affects young, otherwise healthy men, particularly athletes and manual laborers engaged in strenuous overhead activity [2,4-7]. Typical clinical features include acute unilateral upper-extremity pain, swelling, cyanosis and prominent superficial veins [8,9]. Potential complications include pulmonary embolism, recurrent thrombosis and post-thrombotic symptoms that may cause persistent pain, swelling and functional impairment [2,8].

Diagnosis requires a high index of clinical suspicion. Digital pallor or coldness, although not typical of an isolated venous presentation, should prompt assessment for associated arterial involvement [10]. Duplex ultrasonography (DUS) is the usual first-line investigation for suspected upper-extremity DVT. Computed tomography (CT) or magnetic resonance venography can provide additional information regarding thrombus extent and thoracic-outlet anatomy, while dynamic DUS with provocative arm positioning may help assess positional vascular compression [2]. Management should be individualized according to symptom duration and severity, thrombus extent, bleeding risk and evidence of anatomical compression. Anticoagulation is a principal component of treatment, while catheter-directed thrombolysis (CDT) and thoracic-outlet decompression by first-rib resection (FRR) may be considered in selected patients with acute PSS [2,11,12].

This report describes PSS in a 46-year-old woman who was neither an athlete nor a manual laborer, but whose symptoms developed following a repetitive overhead activity. The clinical, imaging and operative findings were consistent with PSS associated with thoracic-outlet compression, accompanied by episodic digital pallor and focal subclavian arterial narrowing. The case highlights the importance of considering PSS outside its typical demographic, the complementary roles of vascular imaging and the possibility that arterial imaging abnormalities may accompany a predominantly venous presentation. The hemodynamic significance of such abnormalities requires careful clinical and imaging correlation.

## Case presentation

A 46-year-old woman presented with a four-day history of right upper-limb pain and swelling that developed following a routine gym session involving dumbbell overhead-press exercises. She initially experienced discomfort and stiffness in the right shoulder, which gradually progressed to swelling and intermittent bluish discoloration of the arm, particularly upon elevation. She also reported episodic pallor affecting the thumb and index finger. She was a non-smoker and had no history of malignancy, central venous catheterization, estrogen-containing contraception or hormone therapy, known thrombophilia, or personal or family history of venous thromboembolism. There was no recent trauma, surgery or immobilization, and she was neither pregnant nor postpartum.

On physical examination, the right upper limb was mildly swollen, with a prominent superficial vein over the shoulder region. When both arms were raised above the head, the right arm developed bluish discoloration within 10-15 seconds, while the right radial pulse remained palpable. Peripheral pulses were otherwise symmetrical and palpable, and the neurological examination was normal. Baseline laboratory investigations showed a D-dimer of 260 ng/mL, platelet count of 315 × 10^9^/L, international normalized ratio of 1.1, prothrombin time of 11.4 seconds, activated partial thromboplastin time of 22.9 seconds and activated partial thromboplastin time ratio of 0.9. Urea, creatinine and electrolytes were within the local reference ranges.

Initial DUS identified right subclavian vein thrombosis, while the internal jugular, axillary and brachial veins were reported as patent and compressible (Figure 1). CT venography (CTV) performed the following day showed an approximately 6.5-cm filling defect involving the right axillary and subclavian veins (Figure 2). These sequential imaging studies were therefore discordant regarding axillary vein involvement. CT angiography (CTA) additionally revealed focal “pinhole” stenosis of the right subclavian artery at the thoracic outlet, with the remainder of the artery reported as widely patent (Figure 3). Together with the episodic digital pallor, this suggested an associated arterial compressive component, without distal arterial thrombosis.

**Figure 1:**
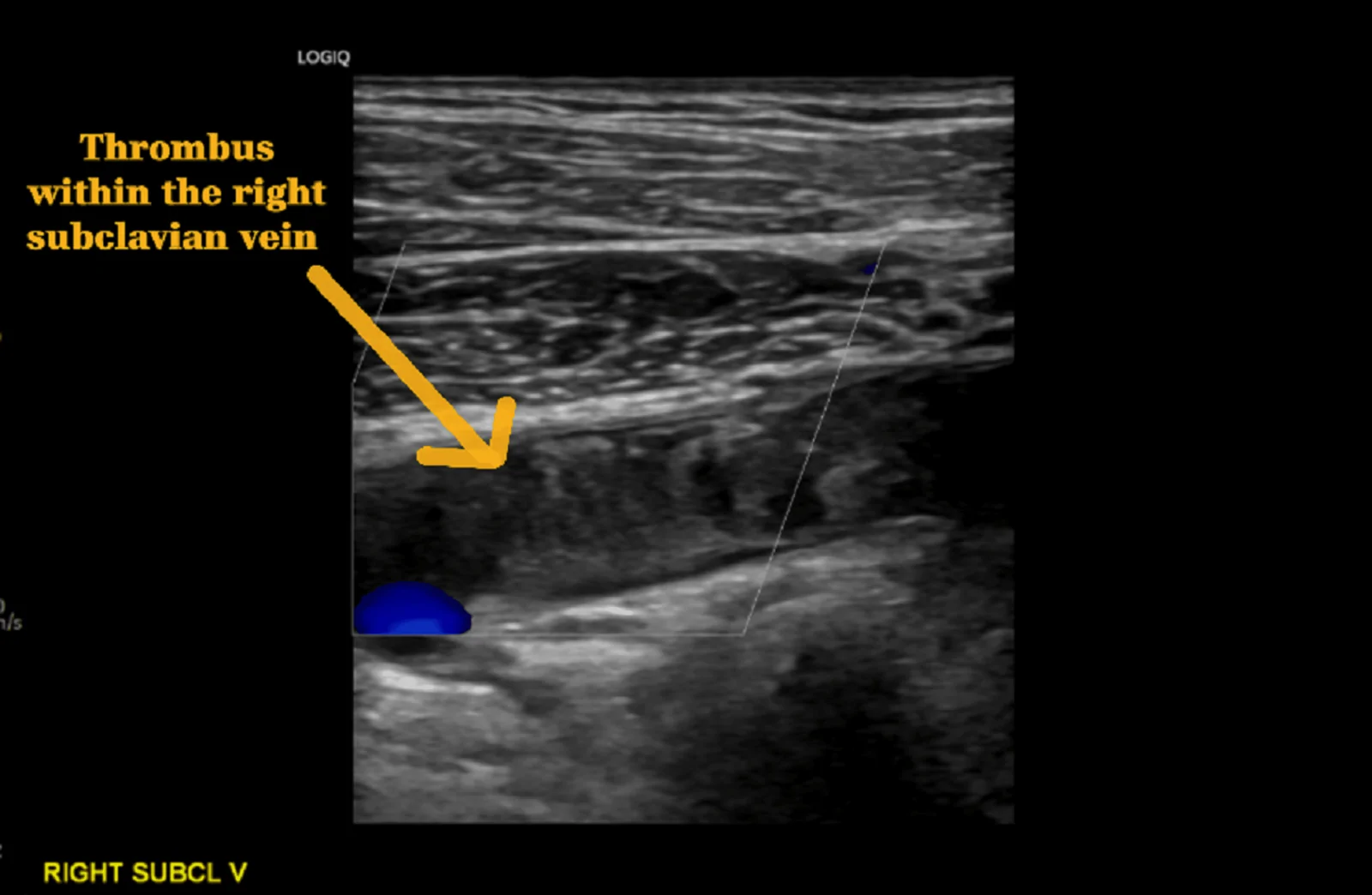
Duplex ultrasonography demonstrating right subclavian vein thrombosis The arrow indicates a thrombus within the right subclavian vein.

**Figure 2:**
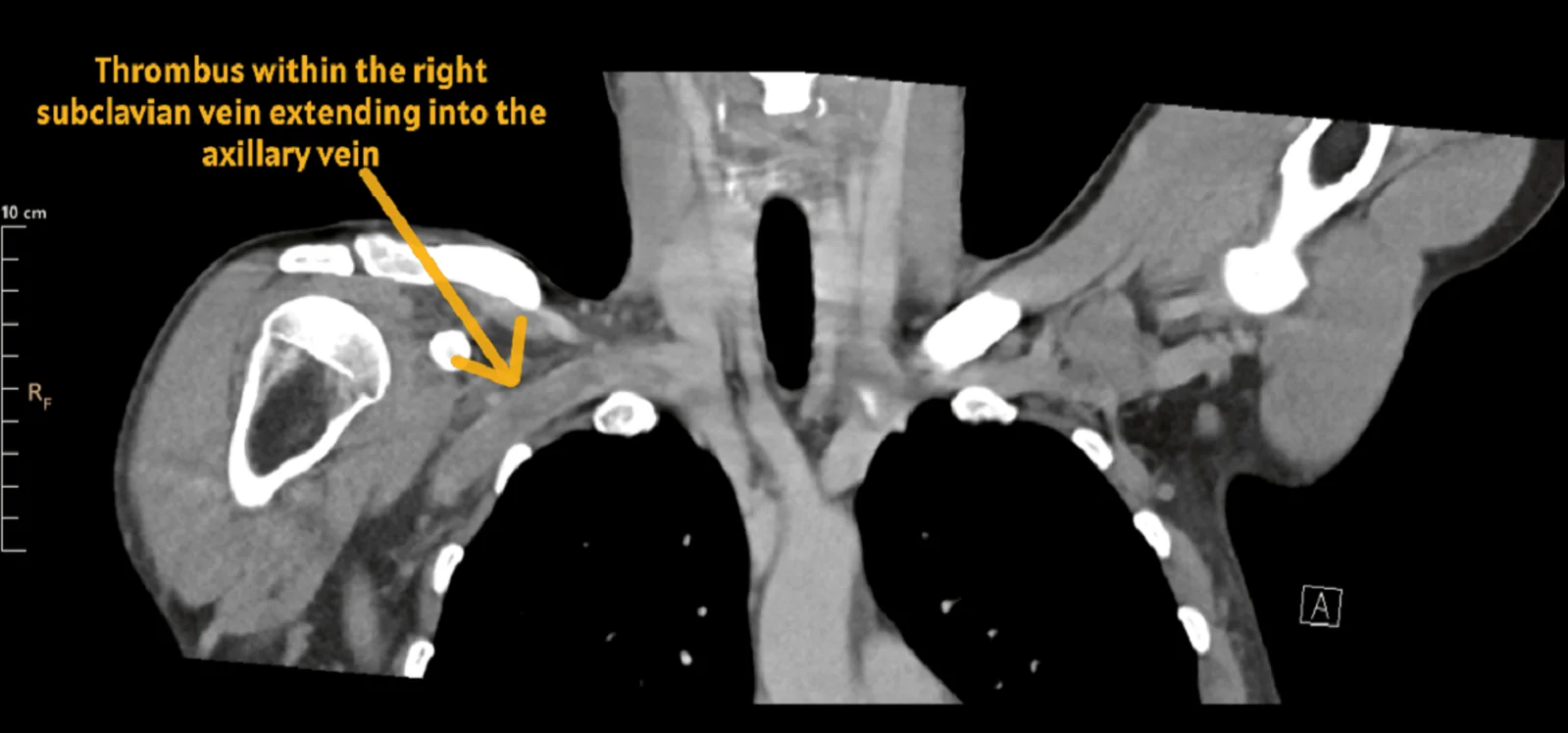
CT venography demonstrating right axillosubclavian vein thrombosis The arrow indicates a thrombus involving the right axillary and subclavian veins.

**Figure 3:**
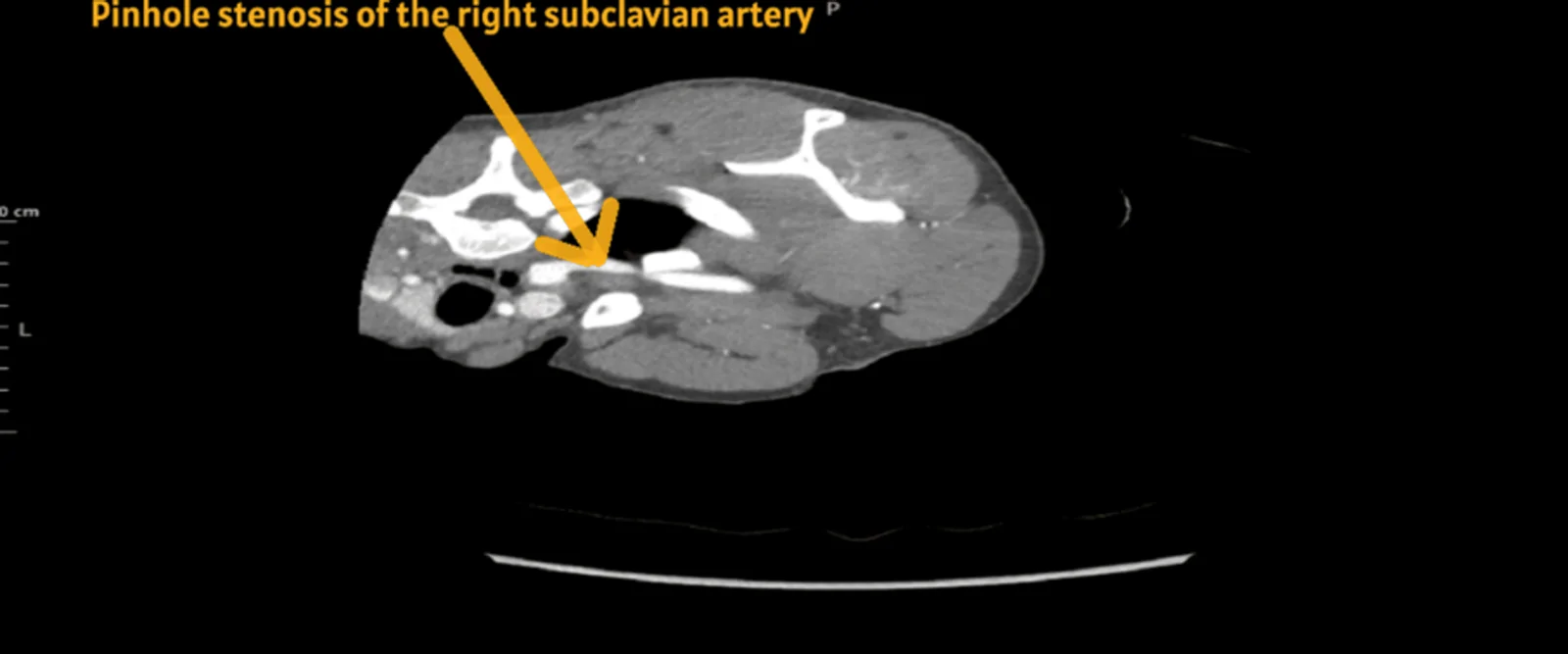
CT angiography demonstrating focal right subclavian arterial stenosis The arrow indicates focal “pinhole” stenosis of the right subclavian artery at the thoracic outlet.

The patient was referred to the vascular team and underwent CDT using a total of 6 mg of alteplase with adjunctive suction thrombectomy and balloon angioplasty. Axillary vein patency improved; however, the distal subclavian vein remained occluded. Intravenous heparin was administered during the procedure, followed by a continuous heparin infusion while awaiting surgical decompression. FRR was performed the following day through combined supraclavicular and infraclavicular incisions. A prominent scalene tubercle was identified intraoperatively. The anterior scalene was divided at its insertion, and the first rib was resected to provide adequate space for the subclavian vein. The procedure was completed without complications.

Postoperative DUS demonstrated triphasic arterial waveforms and good arterial flow throughout the right upper limb, with no significant stenosis or change between stress and rest positions. The right subclavian vein was patent, with spontaneous respiratory phasic flow and no residual thrombus or overt vascular impingement. She was transitioned to apixaban for a planned duration of three months and discharged with follow-up arranged after four weeks. At review, she reported complete resolution of the swelling, positional bluish discoloration and episodic digital pallor, while repeat DUS showed maintained venous patency without recurrent thrombosis.

## Discussion

PSS is a rare primary axillo-subclavian vein thrombosis associated with extrinsic compression of the subclavian vein at the costoclavicular junction and represents the venous form of thoracic outlet syndrome [2,13]. It has an estimated incidence of 1-2 cases per 100,000 population per year, with a mean age at presentation in the early 30s and a male-to-female ratio of approximately 2:1 [14]. Repetitive or strenuous upper-limb activity may promote endothelial injury and venous stasis, contributing to thrombus formation in accordance with Virchow’s triad [7,15].

This case describes PSS in a 46-year-old woman who developed symptoms following a routine gym session involving dumbbell overhead-press exercises. The diagnosis was supported by right upper-extremity pain and swelling, a prominent superficial vein, rapid cyanotic discoloration during arm elevation and radiological confirmation of axillo-subclavian vein thrombosis. The absence of identified secondary provoking factors, together with the temporal relationship to overhead activity, prompted consideration of a primary process. CTA identified focal subclavian arterial stenosis at the thoracic outlet, with the remainder of the artery reported as widely patent. A prominent scalene tubercle was also observed intraoperatively; however, the extent to which it contributed to the arterial narrowing or venous thrombosis could not be determined.

CDT was performed nine days after symptom onset, followed by FRR the next day and subsequent transition to apixaban. Thrombolysis is generally reported to be more effective during the acute phase, commonly described as within two weeks of symptom onset [2,11]. However, this period should not be interpreted as a strict treatment threshold, and the present case cannot establish the optimal timing of intervention. Observational evidence has reported favorable outcomes with multimodal treatment in appropriately selected patients with PSS. A systematic review and meta-analysis of 60 studies involving 2,653 patients reported persistent symptoms in 46% of patients treated with anticoagulation alone, whereas 96% of those treated with CDT followed by FRR were symptom-free at the end of follow-up. Pooled comparative analyses favored CDT plus FRR over anticoagulation alone (OR 13.89, 95% CI 1.08-179.04), anticoagulation plus FRR (OR 2.29, 95% CI 1.21-4.35), and CDT plus anticoagulation without FRR (OR 8.44, 95% CI 1.12-59.53) [11]. However, these comparisons were based predominantly on retrospective observational studies with a risk of possible inclusion bias and should therefore be interpreted cautiously [11]. Accordingly, they provide context for the combined approach used in this case rather than establishing its superiority for all patients. A 2021 meta-analysis of 25 studies involving 1,511 patients reported thrombus resolution in 78% of patients treated with thrombolysis compared with 41% treated with anticoagulation alone, together with favorable venous patency and symptom outcomes when FRR followed thrombolysis [12]. Lugo et al. similarly reported higher venous patency and symptom-relief rates among patients undergoing FRR than among those in whom the first rib was not removed [16].

The presentation in this case was less typical because it occurred in a 46-year-old woman who was neither an athlete nor a manual laborer and had no previous similar episodes. The routine dumbbell overhead-press exercise may have contributed to the acute thrombotic event, although the available evidence does not establish causation or explain why the event occurred at this age. The focal subclavian arterial stenosis represented an unusual additional imaging finding; however, its hemodynamic significance was not formally established because no preoperative dynamic arterial study or pressure-gradient measurement was performed. A prominent scalene tubercle was also observed intraoperatively, but the extent to which it contributed to the arterial narrowing or venous thrombosis could not be determined.

Following thoracic-outlet decompression, postoperative dynamic DUS demonstrated triphasic arterial waveforms, with no significant residual stenosis or vascular impingement at rest or during stress. The clinical approach involved multimodal imaging with DUS, CTV and CTA, which confirmed axillo-subclavian vein thrombosis and identified associated focal subclavian arterial stenosis. Although the axillary vein was reported as patent on DUS, CTV performed the following day demonstrated axillary-vein involvement. This discrepancy represents an important diagnostic consideration. The available imaging could not determine whether this reflected interval thrombus propagation or incomplete assessment of the axillary vein on the initial duplex examination. In this case, CTV provided complementary anatomical information regarding the extent of thrombosis and informed subsequent management planning. At four-week follow-up after CDT, FRR, anticoagulation and postoperative care, the patient’s symptoms had resolved, and DUS confirmed maintained venous patency with normal phasic flow.

Despite the favorable outcome, certain limitations should be acknowledged. A comprehensive thrombophilia assessment was not performed; therefore, an additional prothrombotic predisposition could not be fully excluded. Nevertheless, the temporal relationship with repetitive overhead activity and the demonstrated thoracic-outlet compression supported the diagnosis of PSS. Longer-term clinical and imaging data were unavailable; therefore, sustained venous patency and late post-thrombotic outcomes could not be assessed. Furthermore, although focal subclavian arterial stenosis was demonstrated radiologically, its hemodynamic and clinical significance was not formally established, and no arterial intervention was required. As this report describes a single patient, its findings cannot be generalized to the broader population with PSS.

## Conclusions

PSS should be considered in patients presenting with acute unilateral upper-extremity swelling, pain, cyanosis or prominent superficial veins, particularly following recent repetitive or strenuous overhead activity. The accompanying digital pallor and focal subclavian arterial stenosis highlight the importance of considering associated arterial abnormalities when evaluating a predominantly venous presentation. However, the available evidence did not establish a causal relationship between the arterial stenosis and thoracic outlet compression. Early diagnosis enabled prompt referral for individualized vascular management. In this case, combined management with anticoagulation, CDT and FRR was associated with symptom resolution and maintained venous patency on DUS at four-week follow-up. Longer-term clinical and radiological assessment is required to evaluate recurrence and post-thrombotic complications.
